# CRISPR/Cas12a mediated knock-in of the Polled Celtic variant to produce a polled genotype in dairy cattle

**DOI:** 10.1038/s41598-020-70531-y

**Published:** 2020-08-11

**Authors:** Felix Schuster, Patrick Aldag, Antje Frenzel, Klaus-Gerd Hadeler, Andrea Lucas-Hahn, Heiner Niemann, Björn Petersen

**Affiliations:** 1 Institute of Farm Animal Genetics, Friedrich-Loeffler-Institute, Hoeltystrasse 10, 31535 Neustadt am Rübenberge, Germany; 2 Department of Gastroenterology, Hepatology and Endocrinology, Hannover Medical School, 30625 Hannover, Germany

**Keywords:** Animal biotechnology, Gene delivery, Molecular engineering

## Abstract

In modern livestock farming horned cattle pose an increased risk of injury for each other as well as for the farmers. Dehorning without anesthesia is associated with stress and pain for the calves and raises concerns regarding animal welfare. Naturally occurring structural variants causing polledness are known for most beef cattle but are rare within the dairy cattle population. The most common structural variant in beef cattle consists of a 202 base pair insertion-deletion (Polled Celtic variant). For the generation of polled offspring from a horned Holstein–Friesian bull, we isolated the Polled Celtic variant from the genome of an Angus cow and integrated it into the genome of fibroblasts taken from the horned bull using the CRISPR/Cas12a system (formerly Cpf1). Modified fibroblasts served as donor cells for somatic cell nuclear transfer and reconstructed embryos were transferred into synchronized recipients. One resulting pregnancy was terminated on day 90 of gestation for the examination of the fetus. Macroscopic and histological analyses proved a polled phenotype. The remaining pregnancy was carried to term and delivered one calf with a polled phenotype which died shortly after birth. In conclusion, we successfully demonstrated the practical application of CRISPR/Cas12a in farm animal breeding and husbandry.

## Introduction

**Figure 1:**
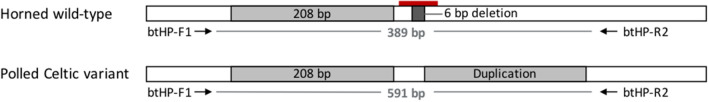
Schematic depiction of the horned locus adapted from Wiedemar et al.^7^. The Polled Celtic (Pc) variant (bottom) consists of a 208 bp duplication in combination with a 6 bp deletion. The CRISPR/Cas12a system targeted the 6 bp sequence of the wild-type (WT) variant (red bar) which is not present in the Pc variant. Specific primers (btHP-F1 and btHP-R2) were employed to distinguish between the WT sequence (389 bp) and the Pc variant (591 bp).

Animal welfare is a crucial aspect of modern animal husbandry. As the global demand for dairy products increases alongside the growth of the population worldwide, animal farming faces new challenges such as increasing food production output without compromising animal welfare and minimizing the environmental footprint^1^. In today’s dairy cattle farming the vast majority of cows display a horned phenotype. This, however, poses an increased risk of injury for the animals themselves as well as for farmers, hence a polled phenotype is preferred^2,3^. Naturally polled phenotypes mainly exist in beef breeds such as Angus. It was reported that in some breeds such as Holstein–Friesian (HF) the polled population originates from only two breeding bulls^4^. This demonstrates the necessity to increase the genetic pool of polled individuals within these breeds. Previous studies revealed the genetic background of polledness^5–8^. Two genetic variants within the *polled locus* on chromosome 1 are known to cause the polled phenotype. One variant is the Celtic mutation (Polled Celtic, Pc) located within an intergenic region of chromosome 1 of the bovine genome (*horned locus*). This autosomally inherited structural variant consists of a complex 202 bp insert-deletion (indel) mutation on bovine Chromosome 1 which has been described before (Fig. 1). Another causative mutation is the *Polled Friesian* (Pf) variant which is the only polled variant present in dairy cattle and consists of an 80 kb duplication accompanied by three single nucleotide polymorphisms (SNPs). More recently, further genetic variants (*Polled Mongolian* and *Polled Guarani*) within the same locus were identified which also lead to a polled phenotype^9^. All known variants connected to polledness are collected in the OMIA database (www.omia.org).

The application of modern breeding systems together with advanced knowledge about the bovine genome facilitated breeding for polledness^10–12^. In the past, however, the selected high-performance bulls were mostly horned and the breeding towards polledness was not considered relevant by farmers. Therefore, the distribution of the polled trait by classical cross-breeding is complicated by poor breeding and production properties of polled HF cattle as well as small gene pools of polled dairy cattle. The introgression of polledness into the genome of a valuable breeding bull without negatively affecting other important traits is estimated to take four to eight generations^11,13^.

Novel programmable DNA nucleases such as zinc finger nucleases (ZFNs), transcription activator-like nucleases (TALEN) and clustered regularly interspaced short palindromic repeats/CRISPR-associated protein 9 (CRISPR/Cas9) system became widely applicable across various species^14^. As the newest member of the family, CRISPR/Cas9 has proven to be highly efficient, cheap in its production and easy in handling^15^. For the application in eukaryotic organisms, an RNA complex was designed to form a single guide RNA (gRNA) which guides the Cas9 nuclease to the respective target sequence (i.e. RNA to DNA binding)^16,17^. The gRNA can be re-programmed to target virtually any genomic locus, however, a protospacer adjacent motif (PAM) sequence at the 3′-end of the gRNA (5′-NGG-3′) is necessary for binding and cleavage of the DNA^18,19^. Other CRISPR/Cas systems, such as the CRISPR/Cas12a (formerly Cpf1), were designed to overcome this PAM-restriction^20^. CRISPR/Cas12a is a type V class II CRISPR system that only requires a single CRISPR RNA (crRNA) instead of a tracr:crRNA complex like CRISPR/Cas9. Furthermore, a 5′-TTTN-3′ PAM sequence upstream of the gRNA is required for binding and cleavage of the target DNA. This makes CRISPR/Cas12a a suitable alternative for T-rich loci. Another useful feature of CRISPR/Cas12a is its distinct cleavage pattern. CRISPR/Cas9 possesses two cleavage domains (HNH and RuvC) and cutting of the target sequence results in blunt ends, whereas Cas12a possesses only one active cleavage domain in the site of the RuvC domain which cuts one DNA in cis- and the other in trans-position^21^. This leads to the generation of two non-homologous overhangs (i.e. sticky ends)^22^. These sticky ends facilitate the introduction of new DNA sequences, which makes the CRISPR/Cas12a system a promising option for knock-in experiments^23^.

A valuable method for implementing genome editing in cattle is the somatic cell nuclear transfer^24^. It allows the in vitro production of genetically modified somatic cell lines such as fibroblasts which can be characterized prior to being employed as donor cells for the somatic cell nuclear transfer^25,26^. In a previous experiment, the Pc variant was introgressed into dairy cattle using TALEN; the generated offspring showed a polled phenotype^27,28^.

Here, we established the CRISPR/Cas12a system as a novel method to integrate the Polled Celtic variant into the genome of a horned HF breeding bull to produce offspring with a polled phenotype and thereby rendering dehorning unnecessary.

## Results

### Generation of knock-in cell lines

**Figure 2:**
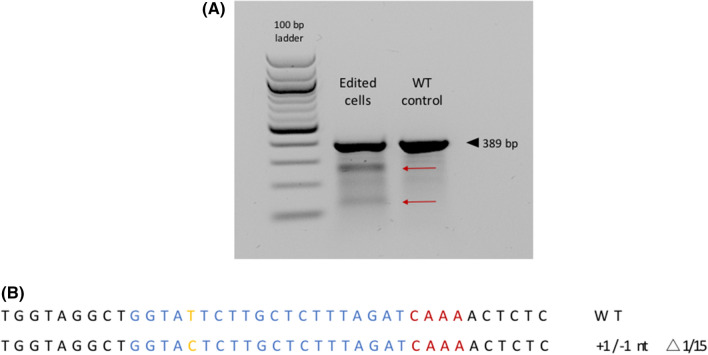
Assessment of nuclease activity. The T7 endonuclease-I assay showed additional bands (red arrows), indicating cleavage of the wildtype sequence (**A**). Sub-cloning of the target sequence (blue: gRNA, red: PAM sequence). One out of 15 sub-clones (∆1/15) reveal indel formation (**B**).

A gRNA for the CRISPR/Cas12a system was designed to target the wildtype sequence of the horned locus (Fig. 1). In a first experiment to check the capability of the CRISPR/Cas12a to target the horned locus, adult fibroblasts derived from a horned Holstein–Friesian bull (total merit index 141) were co-transfected with plasmids expressing CRISPR/Cas12a and gRNA, but without an HDR template carrying the polled Celtic variant. The T7 endonuclease-I assay of lysed cells showed cleavage of heteroduplex DNA resulting in two additional bands, which indicate the on-target efficiency of the transfected nuclease (Fig. 2A). Additionally, PCR amplicons of the transfected cells were sub-cloned and sequenced. A 1 ± 1 nt indel mutation at the predicted cutting site confirmed the nucleases specificity to introduce an on-target DSB (Fig. 2B).

**Figure 3:**
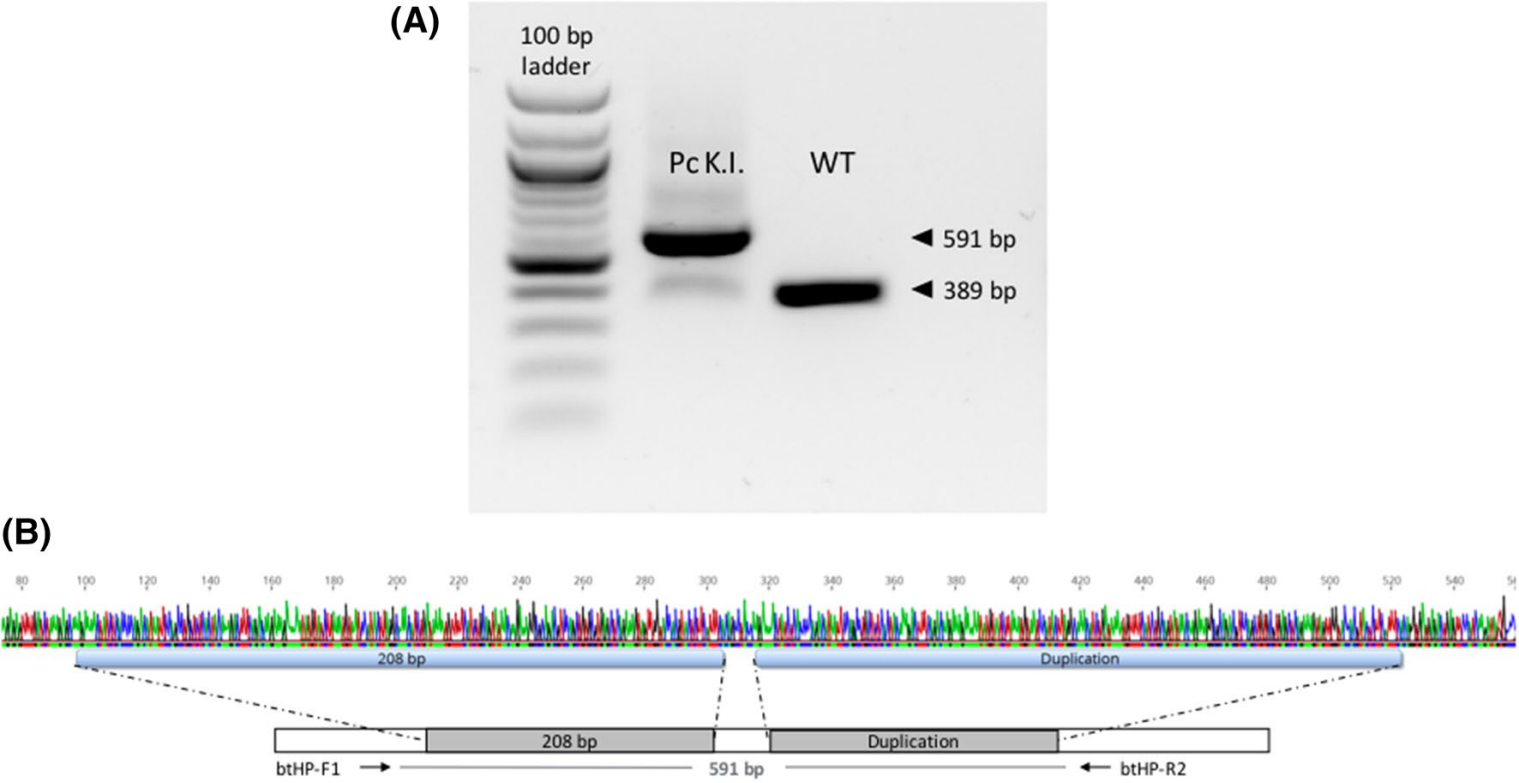
In vitro generation of Polled Celtic knock-in (Pc K.I.) cell line. PCR analysis of edited cell line (Pc K.I.) with Pc-specific primers (**A**). The PCR fragment of the edited cell line (591 bp) was 202 bp larger than the WT fragment (389 bp). Sanger sequencing revealed the integration of the Pc variant (**B**).

Subsequently, wildtype fibroblasts from the same bull were co-transfected with CRISPR/Cas12a, gRNA and HDR template for the polled variant. PCR analysis initially showed that only a small proportion of cells carried the desired knock-in. The exact knock-in efficiency could not be assessed directly after transfection since the PCR could not distinguish between the genomic DNA and the HDR template which might have led to false positive results. Using single-cell dilution, we were able to generate a population of knock-in positive cell clones (Pc K.I.) which then served as donor cells for SCNT (Fig. 3A,B).

### Somatic cell nuclear transfer and embryo transfer

Pc K.I. cell clones were used as donor cells for SCNT. A total of 70 clones were produced of which 66 were successfully fused. Sixty-four embryos showed cleavage on day 5 of in vitro culture of which 18 embryos developed to expanded or hatched blastocysts on day 7 of in vitro cultivation (IVC) (28.1% blastocyst rate). One or two expanded (hatched) blastocysts were transferred into nine synchronized recipients after seven days of IVC (in total 12 cloned embryos, 3 recipients received 2 embryos). The remaining embryos served as a quality control and were maintained in culture until day 8, six showed delayed development. These were not transferred, resulting in a final blastocyst rate of 37.5% (Table 1). Six recipients initially established a pregnancy as determined by ultrasound diagnostic on day 40 of gestation (66.7%). Four animals could not maintain pregnancy past the first trimester. One cow was sacrificed on day 90 of gestation in order to analyze the fetus. The remaining pregnancy was carried to term.

**Table 1:** Cloning of Pc K.I. fibroblasts.

Maturation rate of oocytes (%)	Donor cell line for SCNT	SCNT complexes built (n)	Fusion rate (%)	Cleavage rate, day 5	Blastocyst rate, day 7	Blastocyst rate, day 8
74/160 (46.3% )	Pc K.I	70	66*/70 (94.3%)	51/64 (79.7%)	18/64 (28.1%)	24/64 (37.596)

Animal number	Transferred embryos	Pregnancy				
5,647	1 hatched blastocyst	Yes				
5,628	1 hatched blastocyst					
5,668	1 expanded blastocyst, 1 blastocyst	Yes*				
5,659	1 hatched blastocyst	Yes				
5,670	1 hatched blastocyst					
5,669	1 hatched blastocyst	Yes**				
5,716	2 blastocysts	Yes*				
5,671	1 expanded blastocyst, 1 early blastocyst	Yes*				
5,732	1 hatched blastocyst					

Top: Day 7 embryos were used for embryo transfer. Remaining embryos were left in culture, six embryos showed delayed development to blastocysts.

*Two fused complexes were lost during handling. Bottom: One to two embryos were transferred per animal. *Pregnancy could not be maintained past day 90 of gestation.

**Pregnancy was terminated on day 90 of gestation for analysis of the fetus.

### Genomic and phenotypic analysis of the fetus

**Figure 4:**
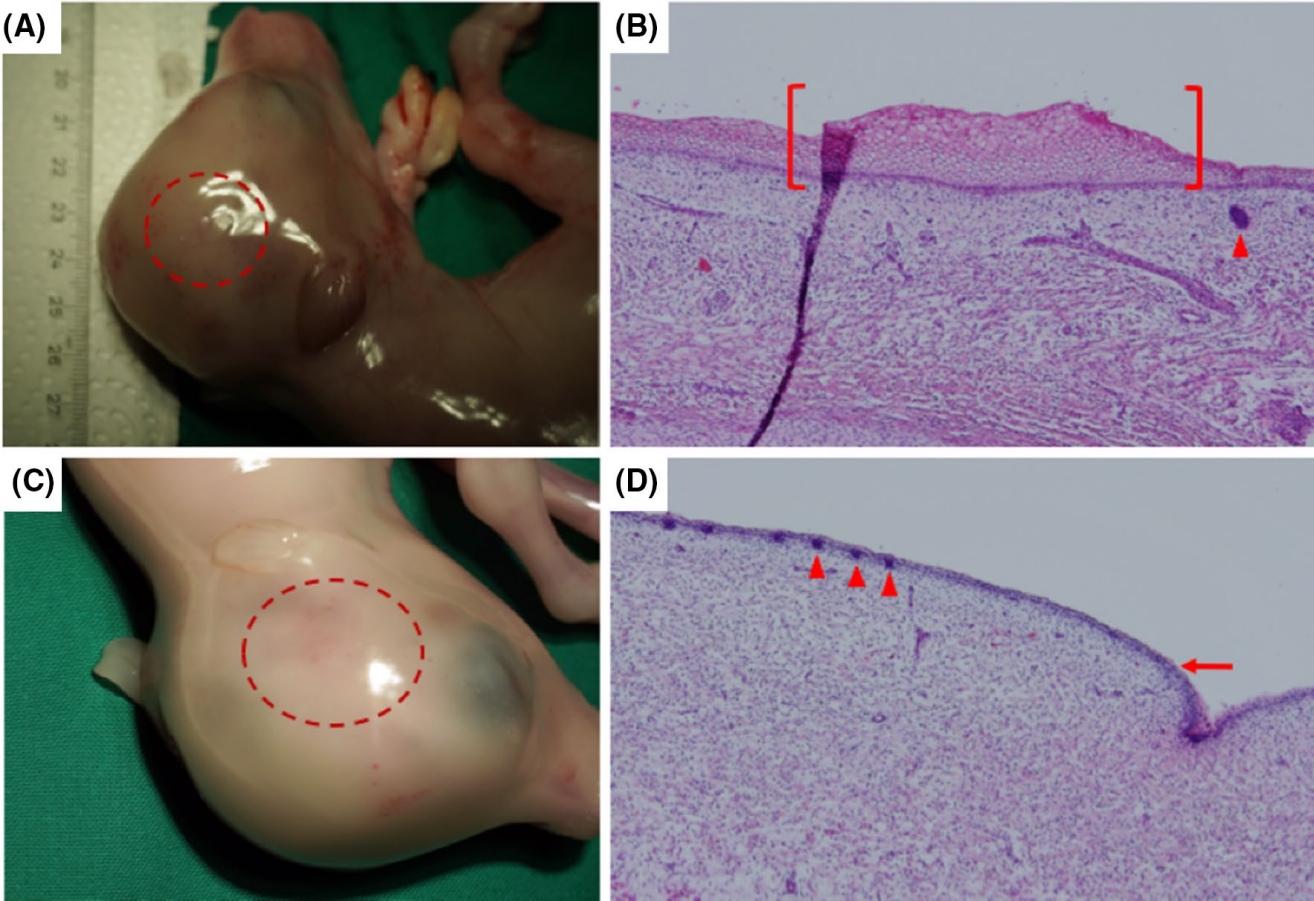
Phenotypic analyses of fetuses. A horned fetus was collected from a slaughterhouse (**A**). The fetal horn bud is circled in red. The corresponding histological analysis of the frontal skin (**B**) revealed a thickened epidermis with 11 to 13 layers of vacuolated keratinocytes (red bracket). Fetal hair follicles (red triangle) were not detected in dermal layers beneath the fetal horn bud. The fetus cloned from Pc K.I. fibroblasts (**C**) showed a phenotypically smooth frontal skin (red circle). Its histological examination (**D**) revealed no thickened epidermis with only one to three layers of keratinocytes (red arrow). No fetal hair follicles (red triangles) were detected in dermal layers beneath this area (histological images were acquired in 40-fold magnification).

Potential horn buds can be detected at an early stage of development^30^. In the *horned WT* control, horn buds were detected macroscopically (Fig. 4A). Histological analysis of the frontal skin revealed thickening of the epidermis with 11–13 layers of vacuolated keratinocytes (Fig. 4B). No fetal hair follicles were detected in the dermal layers beneath the horn bud. In the fetus carrying the Pc variant, no horn buds were detected macroscopically (Fig. 4C). The histological examination showed only slight epidermal thickening with two to three layers of vacuolated keratinocytes (Fig. 4D). No hair follicles were detected in the area of a potential horn bud. Taken together, the fetus was phenotypically polled.

**Figure 5:**
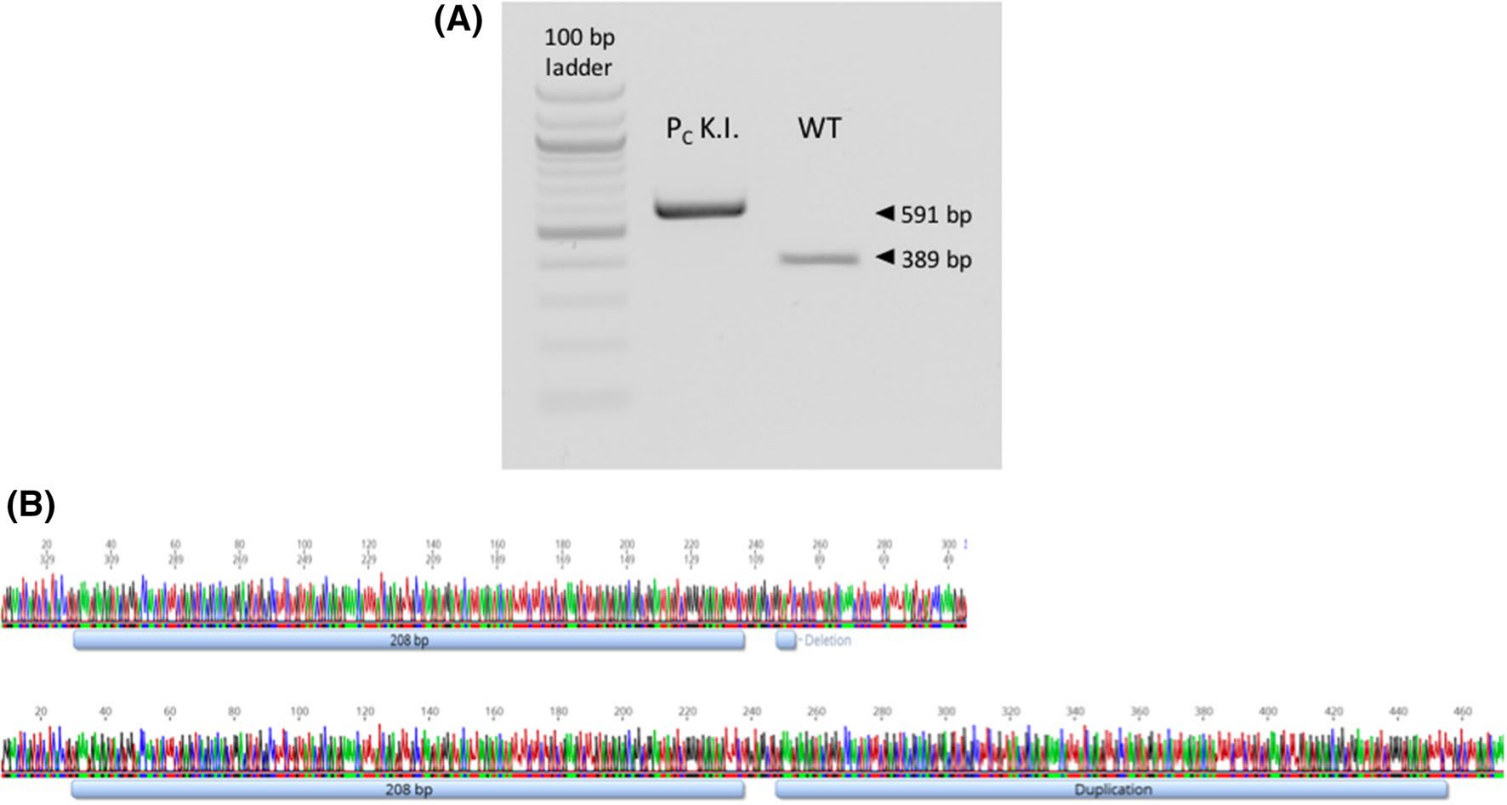
Genomic analysis of fetus Pc K.I. PCR analysis with Pc-specific primers (btHP-F1 and btHP-R2) revealed the integration of the Pc variant into the HF genome (**A**). DNA from the horned HF donor bull served as the wildtype control. Sanger sequencing chromatograms (**B**) show horned variant of the donor bull (top) and the Pc variant of the edited fetus (bottom).

Fetal liver tissue was used to isolate DNA. PCR analysis showed the successful integration of the Celtic mutation (Fig. 5A), the fibroblast donor served as wildtype control. Sanger sequencing confirmed the bi-allelic integration of the Pc variant (Fig. 5B).

#### Delivery of living offspring

**Figure 6:**
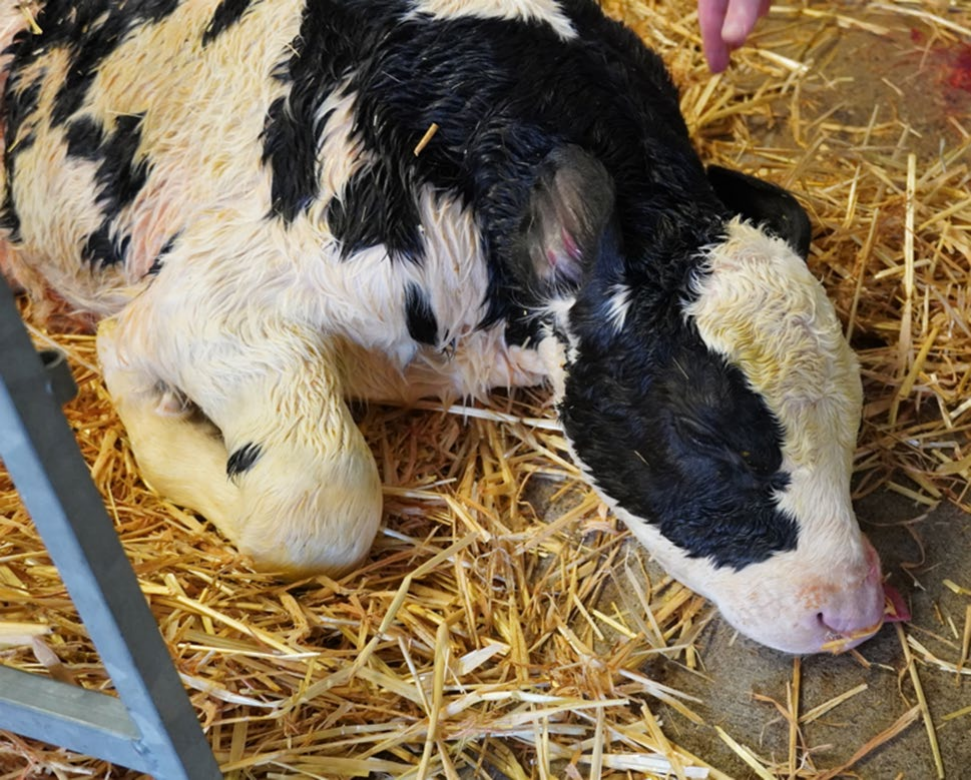
Delivered polled calf. The calf was successfully delivered via caesarian section. The calf died shortly after birth due to multiple organ malformations finally resulting in cardio-vascular failure.

The remaining pregnancy was successfully delivered via caesarian section (Fig. 6). The calf showed a polled phenotype and the genomic analyses were identical to the previously generated fetus as the Pc variant was detected via PCR and Sanger sequencing (Supplement 5). However, the calf diseased on the day of birth. The calf showed an increased birth weight of 78 kg. Its pathological examination revealed malformations of some internal organs including the liver, heart, diaphragm, lungs and skull, finally resulting in acute cardio-vascular failure.

### Vector integration and off-target analysis

Three potential off-target binding sites showed no undesired mutations in the respective loci according to the T7 endonuclease I assay and Sanger sequencing (Supplement 7). During the generation of the knock-in cell line, three plasmids carrying an ampicillin expression cassette were transfected into the bovine fibroblasts. To test for random plasmid backbone integration, PCRs specific to the antibiotic resistance cassette and selected plasmid fragments were conducted (Supplements 2, 3). A faint signal only for the resistance cassette was detected. PCR analysis specific to the HDR template (HP1748-F1 and HP1748-R1) indicates potential integration of another copy of the Pc variant.

## Discussion

Polled cattle are easier and safer in handling and the disbudding of calves contradicts animal welfare, therefore genetically polled cattle are preferred in the dairy industry^2,31^. The Polled Celtic (Pc) variant, a 202 bp indel variant within the *polled locus*, causes a polled phenotype in many beef breeds^5,32^. We hypothesized that the CRISPR/Cas12a mediated knock-in of the Pc variant into the genome of a polled HF breeding bull causes a polled phenotype in offspring produced via somatic cell nuclear transfer (SCNT) within one generation.

In a similar study, the knock-in of the Pc variant into bovine fibroblasts by transfecting TALEN in form of mRNA together with an HDR template carrying the 202 bp indel mutation resulted in cloned offspring with a polled phenotype^27^. In our project, we employed the CRISPR/Cas12a system as a novel and efficient method for the introduction of the Pc variant into the genome of a high-performance HF breeding bull.

The CRISPR/Cas12a nuclease (formerly CRISPR/Cpf1) is a class II CRISPR/Cas system which requires a 5′-(T)TTN-3′ PAM sequence for targeted DNA binding and cleavage^20^. This feature makes it a favorable option in T-rich target regions, such as the target region in the *polled locus*. Another benefit of CRISPR/Cas12a is its distinct DNA cleavage pattern. Contrary to Cas9, Cas12a possesses only one cleavage domain (RuvC-like endonuclease domain) which cuts each DNA single-strand at different sites, resulting in non-homologous 4–5 bp overhangs. This facilitates the correct integration of new DNA sequences independently from the cell cycle and the employed repair mechanism. These characteristics of the CRISPR/Cas12a made it the nuclease of choice for the knock-in of the Pc variant. Here, 20 bp long gRNAs were used, however an updated version of the online tool CRISPOR (https://crispor.tefor.net) predicted higher on-target efficiencies with gRNAs of 24 bp length. An experiment comparing both gRNA lengths could potentially optimize on-target efficiencies by assessing their respective ability to create indel formation and thereby facilitate the knock-in. However, quantitative knock-in efficiencies could not be assessed directly after transfection since the employed assays could not discriminate between cellular DNA and residual template DNA.

SCNT is a crucial step in the generation of offspring from in vitro edited fibroblasts. Following SCNT, the developmental rates of the reconstructed embryos are significantly lower compared to IVF embryos^33^. We reliably produced cloned gene-edited embryos with blastocyst rates of 28.1% on day 7 of cultivation and 37.5% on day 8 (Table 1). In vitro produced embryos were transferred into nine recipients and two pregnancies could be maintained until day 90 of gestation. Embryonic and early fetal death is more frequent in pregnancies generated with cloned embryos^34–37^. Several factors are thought to be involved in decreased full-term development rates. Incomplete de-methylation and subsequent aberrant DNA methylation profiles between embryos produced by in vitro fertilization (IVF) and SCNT have been observed and could deteriorate the developmental capacity of the embryos^38^. This impaired epigenetic reprogramming potentially leads to aberrant gene expression, thereby interfering with the full-term development of respective offspring. Another factor that contributes to fetal mortality could be insufficient placentation. In our study, abnormal placentation was detected via sonography in pregnancies which did not develop full term. Placentomes develop from both the inner cell mass (ICM) and the trophectoderm (TE) of the embryo^39^. It was previously revealed that bovine SCNT embryos showed an increased ICM: total cell ratio (i.e. reduced amount of trophectoderm cells) which may lead to insufficient placentation with subsequent deficits of fetal nourishment^40^.

In our project, one pregnancy was terminated on day 90 of gestation for analysis of the fetus. No fetal horn buds were detected macroscopically. Histological examination of the frontal skin also revealed the absence of a thickened epidermis with additional layers of keratinocytes. These findings are in accordance with a previous study in which the horn status of different fetal stages was examined^30^. Taken together, the phenotypic analyses showed a polled phenotype of the fetus cloned from the Pc K.I. cell line. The remaining pregnancy was successfully delivered via caesarian section. The living offspring also showed a polled phenotype; however, it was not viable on the long term due to organ malformation and showed an increased birth weight of 78 kg. This likely represents artefacts of the cloning procedure, which was commonly described as “large offspring” or “abnormal offspring syndrome (type III AOS)”^41–43^. However, whole genome sequencing would be necessary to entirely disprove a detrimental effect of the genome editing process itself.

Genomic analyses of the fetus confirmed the integration of the Pc variant. However, the exact pathway of how the Pc variant causes polledness is still unknown. One hypothesis is that this mutation affects the expression of micro RNAs (miRNAs) which are not yet annotated. MiRNAs can interact with mRNA which might lead to translational repression or mRNA deadenylation^44–46^. It was also reported that miRNAs are capable of silencing genes or inducing transcription by directly binding to promoter regions of genes^47–49^. Therefore, alteration of miRNA expressing regions might significantly affect gene expression patterns. A novel hypothesis is that Pc and Pf are located in *boundary regions* of topologically associating domains (TADs). TADs are structural subunits of the genome which form loop structures within a chromosome and thereby bringing loci which are usually far apart along the genome into the vicinity of each other, enabling interaction between enhancing regions and promotor regions^50,51^. TADs are separated from *boundary regions* which consist of hundreds to thousands of non-coding nucleotides. Mutations in boundary regions may lead to partial fusion or a shift of their position. This might induce a functional change of the three-dimensional chromatin structure^52–54^. Taken together, these epigenetic frameshifts establish novel enhancer-promotor interactions affecting gene expression in loci which are not located near the causative mutation. To confirm either hypothesis, a refined annotation of the *polled locus* and its associating regions including respective gene expression is necessary.

Further genomic analyses indicated a potential integration of a second copy of the HDR template (Supplement 4). In-depth sequencing of the entire locus or quantitative PCR approaches such as digital PCR will confirm or exclude unintended vector integration. In a previous study in which the Pc variant was introduced into the genome of an HF bull via TALEN, a recent publication revealed the unintended integration of the entire HDR template, including another copy of the Pc variant^29,55^. Even though this undesired event did occur, it did not have a negative effect on the generated animals and this unintended plasmid integration could be corrected by classical cross-breeding^56^.

In conclusion, we successfully established the CRISPR/Cas12a system as a novel method to introduce the Pc variant into the genome of a superior Holstein–Friesian bull and could thereby address current issues in today’s farm animal housing and breeding. Analysis of the generated fetus showed a polled phenotype. Finally, we successfully delivered a polled calf which also showed the CRISPR/Cas12a mediated knock-in of the Pc variant.

## Materials and methods

### CRISPR/Cas12a and guide RNA expression

The LbCas12a expressing plasmid SQT1665 (Addgene plasmid #78744) was employed in this study. Three different complementary pairs of DNA oligonucleotides targeting the six base pair deletion of the Celtic mutation (Supplement 1) were annealed and cloned into the BsmBI cloning site of the gRNA expressing plasmid (BPK3082, Addgene plasmid #78742). For subsequent knock-in experiments gRNA “*LbCas12a gRNA_4*” was used.

### Generation of HDR template

The Pc variant was amplified via PCR with primers encompassing the 202 bp indel variant and the homologous arms (HP1748-F1: 5′ GGGCAAGTTGCTCAGCTGTTTTTG 3′ and HP1748-R1 5′ TCCGCATGGTTTAGCAGGATTCA 3′; product size polled: 1748 bp, product size horned: 1546 bp). PCR conditions were as following: 95 °C for 2 min followed by 32 cycles of 95 °C for 25 s, 62 °C for 30 s and 72 °C for 120 s. Final elongation was achieved at 72 °C for 5 min. Resulting DNA fragments were purified (Invisorb^®^Fragment CleanUp-Kit, Startec, Germany) and cloned into the pCR^TM^2.1 transfection vector (TA Cloning^®^ Kit with pCR™2.1 vector, Thermo Fisher Scientific) as recommended by the supplier.

### Cell culture and transfection

Bovine fibroblasts were isolated from an ear notch of a commercially used horned Holstein–Friesian bull (total merit index of 141) and cultured in Dulbecco’s modified Eagle’s medium (DMEM) with 1–2% penicillin/streptomycin, 1% non-essential amino acids and sodium pyruvate and 10–30% fetal calf serum (FCS) at 37° C and 5% CO_2_. Passaged cells were transfected using the 100 µl kit of the Neon® Transfection System (Invitrogen, Thermo Fisher Scientific) at 1,800 V, one 20 ms pulse. A total of 10 µg plasmid was transfected, respectively.

### T7 Endonuclease I cleavage assay and Sanger sequencing

In order to asses on-target efficiency, the target sequence was amplified via PCR using P_C_ specific primers (btHP-F1: 5′ GAAGGCGGCACTATCTTGATGGAA 3′; btHP-R2: 5′ GGCAGAGATGTTGGTCTTGGGTGT 3′) under the following conditions: 95 °C for 2 min followed by 32 cycles of 95 °C for 25 s, 62 °C for 25 s and 72 °C for 60 s. Final elongation was performed at 72 °C for 5 min. The essay was conducted as recommended by the supplier.

### Somatic cell nuclear transfer and embryo transfer

The somatic cell nuclear transfer and parthenogenetic activation were performed as previously reported^57^. Adult fibroblasts with integrated Pc variant were used as donor cells. Recipient animals were synchronized via repetitive prostaglandin application (alfaCloprost^®^ forte, alfavet, Germany; 2 ml per animal per injection). One expanded or hatched blastocyst was transferred per recipient, respectively.

### PCR-based genotyping and phenotypic analysis of fetus

P_C_ specific primers (btHP-F1 and btHP-R2, see above) were used for detection of the knock-in as well as sequencing primers. The first pregnancy was terminated on day 90 for macroscopic and histological analysis of the fetus. Histological slices of the frontal skin were stained with hematoxylin and eosin stain. A horned wildtype control of similar size (i.e. similar day of gestation) was collected from a slaughterhouse.

### Off-target analysis and vector integration

For the induction of a double strand break at the target site a gRNA was employed which does not have likely potential off-target binding sites according to CRISPR RGEN Tools (https://www.rgenome.net/cas-offinder/; reference genome: Bos taurus (bosTau7)). A selection of three potential off-target binding sites was analyzed via T7 endonuclease I assay and Sanger sequencing (Supplements 6, 7). Potential vector integration was tested via PCR analysis of plasmid specific fragments from each transfected plasmid (Supplements 2, 3).

### Ethical approval

All animals used in this project were kept and treated according to the German welfare law, the German guidelines for animal welfare and EU Directive 2010/63/EU. The animal experiments were approved by the supervisory authority (Lower Saxony State Office for Consumer Protection and Food Safety (LAVES), AZ 33.19-42502-04-17/2398).

## Supplementary information

Supplementary Legends

Supplementary Information 1.

Supplementary Information 2.

Supplementary Information 3.

Supplementary Information 4.

Supplementary Information 5.

Supplementary Information 6.

Supplementary Information 7.
