# Supplementary material for: CRISPR/Cas12a mediated knock-in of the Polled Celtic variant to produce a polled genotype in dairy cattle
Source: Sci Rep. 2020 Aug 11;10:13570. doi: 10.1038/s41598-020-70531-y (PMC7419524; doi:10.1038/s41598-020-70531-y)
Supplement: Supplementary file 1 — Supplementary Legends [file 41598_2020_70531_MOESM1_ESM.docx]

**CRISPR/Cas12a mediated knock-in of the Polled Celtic variant to produce a polled genotype in dairy cattle**

Felix Schuster^1^, Patrick Aldag^1^, Antje Frenzel^1^, Klaus-Gerd Hadeler^1^, Andrea Lucas-Hahn^1^, Heiner Niemann^2^, Björn Petersen^1*^

# Supplements

Supplement 1: List of oligonucleotides cloned into gRNA-expressing vectors.

Supplement 2: List of primers used in this project.

Supplement 3: Vector integration analysis of fetus Pc K.I.

A) Ampicillin resistance cassette specific PCR analysis (25 cycles). A band was detected in the sample (red triangle). Diluted plasmid suspensions served as positive control. B) SQT1665 specific PCR analysis for detection of fragment Cas12a expression cassette. The tested sequence was not detected in the sample. Diluted SQT1665 served as positive control. C) BPK3082 specific PCR analysis for detection of gRNA expression cassette. The tested sequence was not detected in the sample. Diluted BPK3082 served as positive control.

Supplement 4: HDR template-specific PCR.

An additional lower band in the *fetus Pc K.I. sample* (red triangle) indicates a potential second copy of the Pc variant. (Fragment size for polled variant: 1748 bp; fragment size for horned variant: 1546 bp).

Supplement 5: Genomic analysis of the delivered calf.

PCR analysis with Pc-specific primers (btHP-F1 and btHP-R2) revealed the integration of the Pc variant into the HF genome (A). DNA from the horned HF donor bull served as the wild-type control. The Sanger sequencing chromatogram (B) showed the 202 bp indel formation which is the Pc variant.

Supplement 6: Primers used for the off-target analysis.

The listed primers encompassing the respective potential off-target binding sites were used for the T7 endonuclease I assay and as sequencing primers.

Supplement 7: Off-target analysis.

A) PCR Products with the respective primers (B) for Off-target site 1-3 (OTS-1-3) were Sanger sequenced and additionally employed in a T7 endonuclease I assay (C) to detect off-target events. No off targets were detected in either the obtained fetus or the delivered calf as shown by Sanger sequencing (A) and T7 endonuclease I assay (C).
