# Supplementary figures and images for: CRISPR/Cas12a mediated knock-in of the Polled Celtic variant to produce a polled genotype in dairy cattle
Source: Sci Rep. 2020 Aug 11;10:13570. doi: 10.1038/s41598-020-70531-y (PMC7419524; doi:10.1038/s41598-020-70531-y)

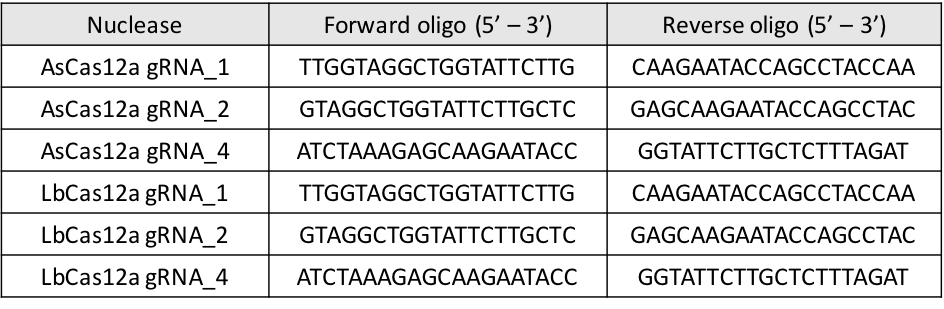

Supplement: Supplementary file 2 — Supplementary Information 1. [file 41598_2020_70531_MOESM2_ESM.png]

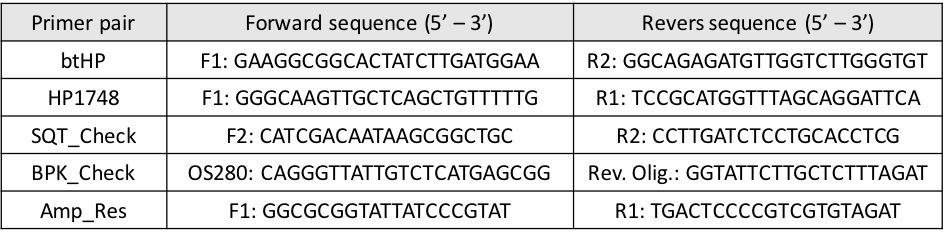

Supplement: Supplementary file 3 — Supplementary Information 2. [file 41598_2020_70531_MOESM3_ESM.png]

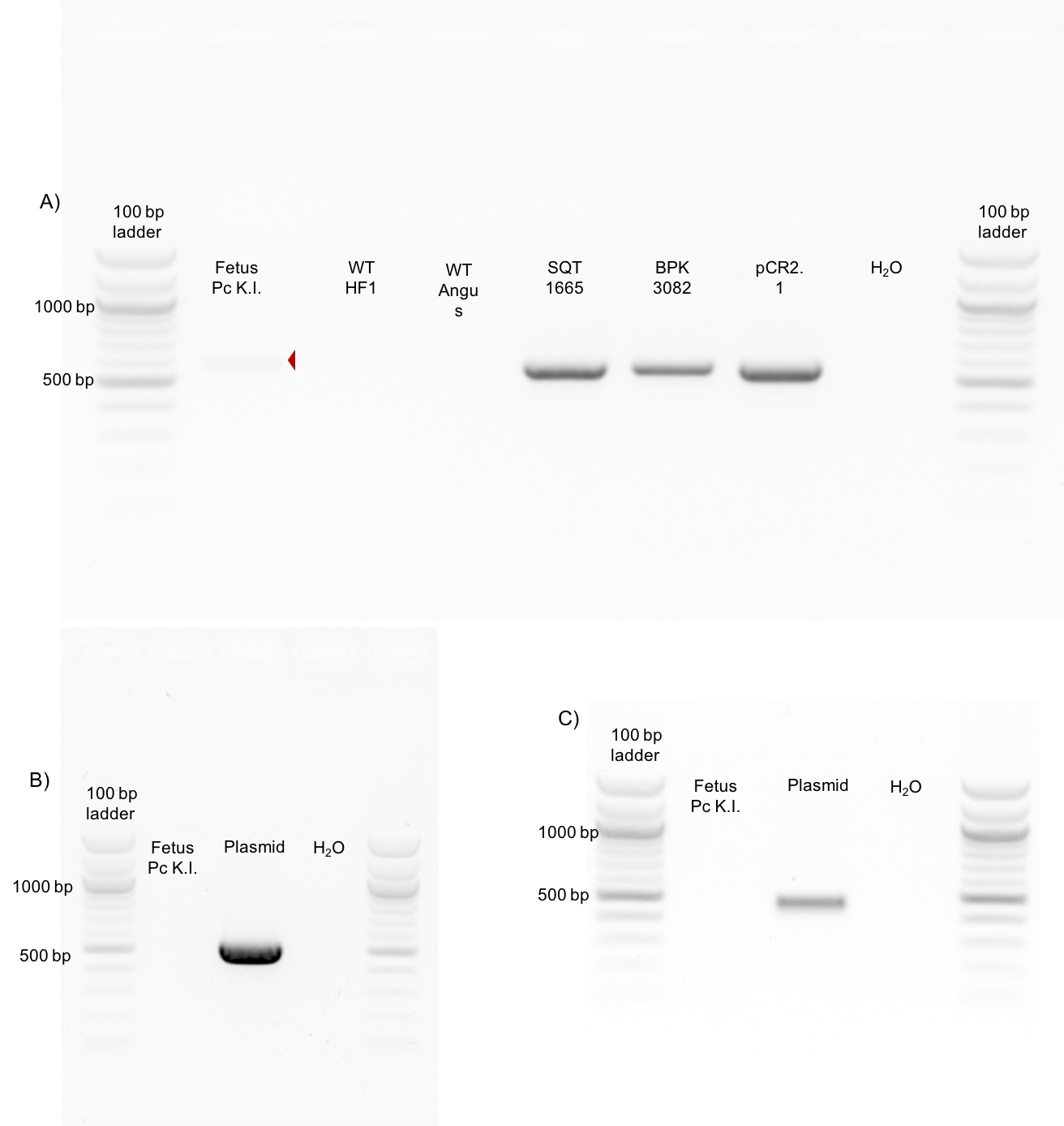

Supplement: Supplementary file 4 — Supplementary Information 3. [file 41598_2020_70531_MOESM4_ESM.png]

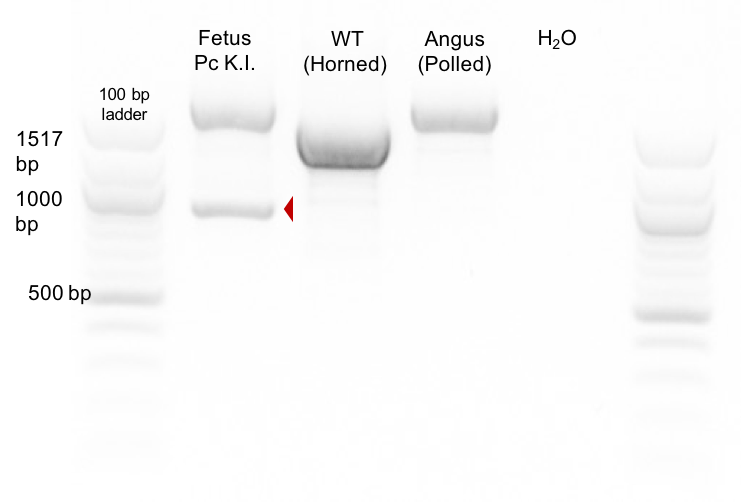

Supplement: Supplementary file 5 — Supplementary Information 4. [file 41598_2020_70531_MOESM5_ESM.png]

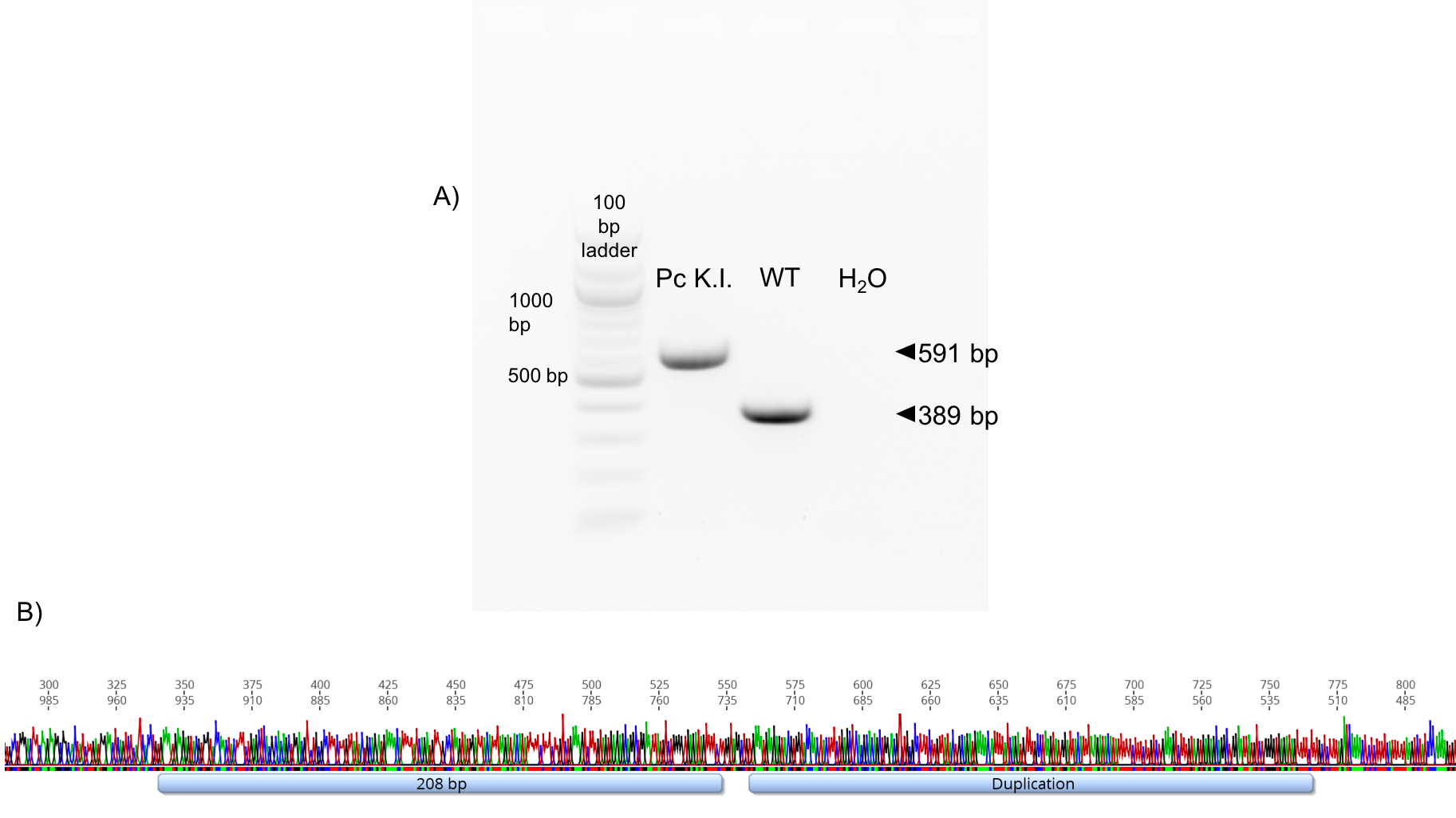

Supplement: Supplementary file 6 — Supplementary Information 5. [file 41598_2020_70531_MOESM6_ESM.png]

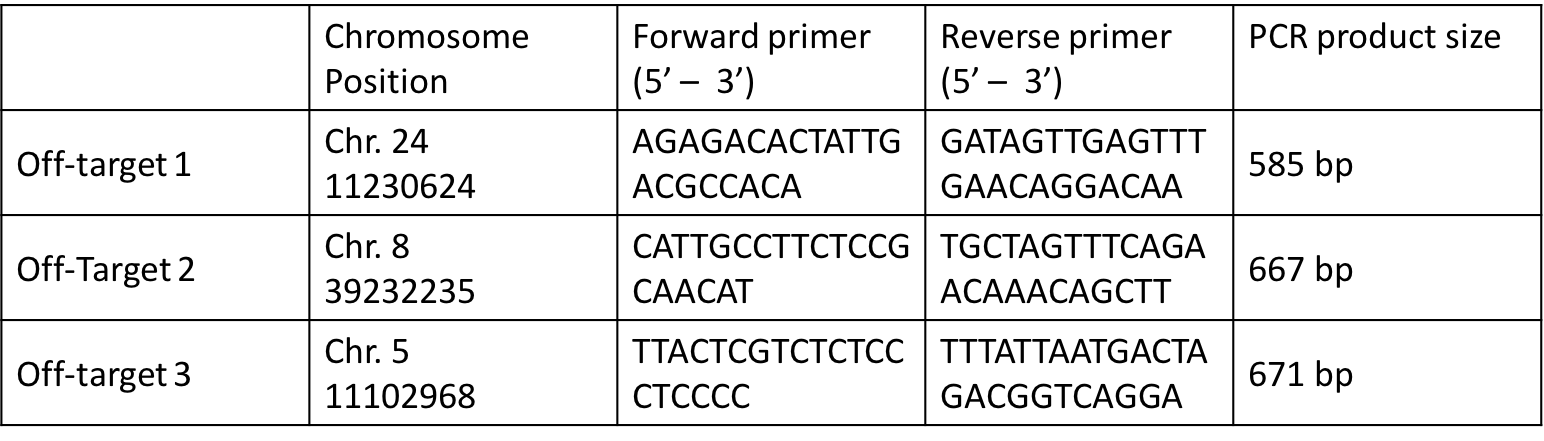

Supplement: Supplementary file 7 — Supplementary Information 6. [file 41598_2020_70531_MOESM7_ESM.png]

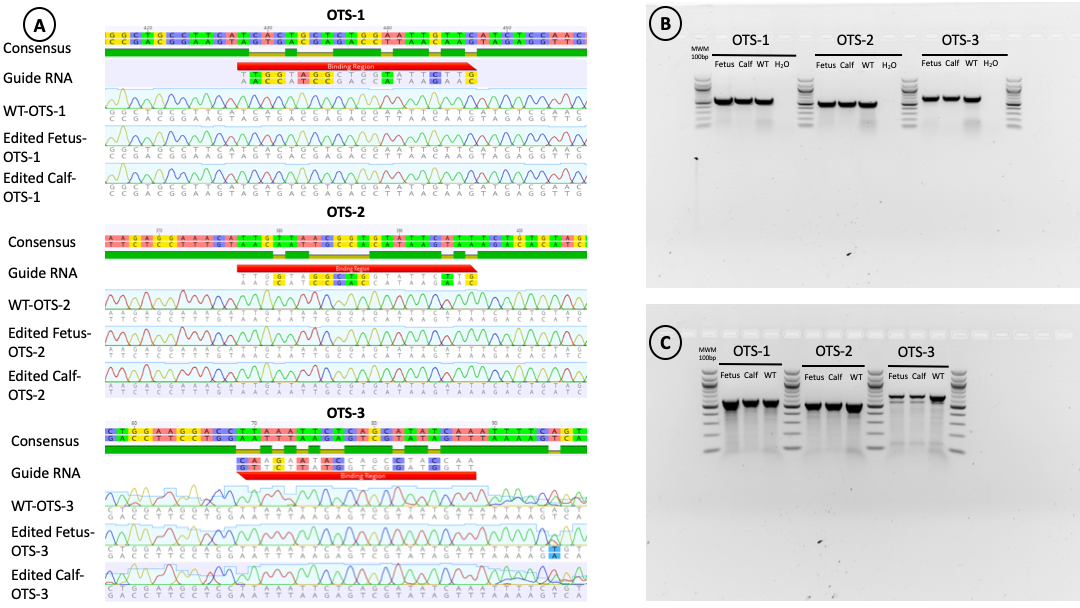

Supplement: Supplementary file 8 — Supplementary Information 7. [file 41598_2020_70531_MOESM8_ESM.png]
